# Prophylaxis in Dentistry

**Published:** 1899-01

**Authors:** D. D. Smith


### PROPHYLAXIS IN DENTISTRY.[1]

BY D. D. SMITH, D.D.S., M.D.

Is the process of disintegration of human teeth known as decay, which so frequently ends in complete destruction, wholly or in part preventable?

Many theories as to the cause or causes of dental caries have been advanced, and many ingenious methods invoked for arresting

---

[1] Read before the Northeastern Dental Society at Hartford, Conn., October 19, 1898.

decay already induced, but nowhere in dental or medical literature is there an attempt to satisfactorily answer this most serious and important question. It confronts the practitioner of medicine and dentistry alike in the mouth of infancy, childhood, youth, adult life, and even down to old age.

Modern medical science, with all its acumen, has thus far seemingly overlooked, as it has totally disregarded, the mouth and teeth as a constant and prolific source of infection in systemic troubles,—gastric, intestinal, cancerous, and pulmonary; and dentistry, engrossed in restoring the ravages of decay, has failed even to discuss, much less earnestly investigate, the subject of dental prophylaxis.

Although interested in dentistry for the past thirty-seven years, and directly in the current of its wonderful progress for thirty-two years consecutively, yet the writer must confess to undivided concentration on the same lines of effort which has characterized the work of the profession in general,—viz., mechanical restoration for teeth already decayed. Recognition of the utter impotency of the present methods of dentistry to cope with the extensive decay in the human teeth, as seen in the mouths of the suffering masses, has more recently stimulated to investigation and effort in the direction of the title of this paper.

Some startling statistics, also, coming to notice, may have had their promptings as the following: Examination of the mouths of the children of the public schools of the city of Toronto by a dentist, the inspections being systematized and extending over a considerable time, revealed that ninety-two per cent. of them were dentally in a pathological state and needing professional attention. A strictly accurate report on the mouths of the children in our public schools and families would, we believe, disclose a pathological condition in excess of that tabulated as existing in Toronto. From unrecorded examinations among the more careful families, extending over a period of years, it is my firm conviction that not three per cent. of the children in the average condition in life, have mouths and teeth in a strictly sanitary and hygienic state. And what has been said of hygienic and pathological conditions in the mouths of children will apply with equal force to the dental outfit of adults.

With these statements before us, let us look briefly at what dentistry is really doing for the community at large,—a matter, perhaps, as well illustrated in our own city of Philadelphia as in any place in the country.

Philadelphia is pre-eminently a city of homes, and has a population of about one and a quarter millions of people. Its houses, *not tenements*, number, it is said, some thousands more than the great metropolis before the consolidation.

It is estimated that only about four hundred thousand of the population receive any benefit at all at the hands of dentistry except the extraction of teeth. Not all of the remaining eight hundred thousand or nine hundred thousand are excluded from the benefits of dentistry through inability; some shut themselves out through fear, others through indifference, and some in inexcusable ignorance, fail of benefits at the hands of the dental profession. It is probably within the bounds of a correct estimate to say that one-half of our population are excluded through pecuniary inability.

The God-likeness of medicine is exhibited in its reaching out in one way or another, often without hope of compensation, to relieve suffering in the humblest of humanity.

Dentistry, in the nature of things, until the State or public bequests shall come to its aid, cannot imitate medicine in this, but we believe it can come to the aid of a suffering race in a most important way and on a grand scale, by presenting methods which are within the reach of the high and the low, for arresting the present rapid destruction of the human teeth. To accomplish anything in this direction there must first be recognition of facts, and then the creation of a literature to make them known.

One of the most recent works on dentistry—one which we ought to be able to point to with pride, "The American Text-Book of Operative Dentistry," a work of six hundred and ninety-one pages of matter, written by fifteen representative men in the profession, and published in 1897; a work of which much that is commendatory may be said, is without a chapter or even a sentence, distinctively as such, on this subject. Illuminated with illustrations of instruments, appliances, methods of operating, good and bad, like other works of its kind, it deals largely with the mechanics of dentistry, and nowhere recognizes the more important matter of the prevention of decay in the teeth.

If we except one or two of the more important and really helpful papers, noticeably a letter in the January, 1898, *Items of Interest*, from Dr. J. L. Williams, of London, and a paper by Dr. I. N. Carr, read before the North Carolina State Dental Society in May last, it may be said that dental prophylaxis has failed to receive the recognition in the journals or before the societies which its importance demands.

The reason why so little interest is manifested in the subject seems obvious. Manufacturers of patent and secret preparations are taking the initiative, and through their wares forcing the matter into notice. Due largely to recommendation and prescription for lack of something better on the part of the medical profession, the public now has in quite general use one of the most inefficient as well as extensively advertised of the self-prescribing nostrums of the present time.

Some of the preparations now in use may be made to supplement in a valuable way the intelligent efforts of the dentist in the direction of prophylaxis, but each and all utterly fail, as they always must, to reach and remedy the *cause* of decay. While comprehensive claims for far-reaching effects upon bacteria and occult diseased conditions of the mouth and teeth are made for many, they bring but little comfort to the true investigator.

In the *Hospital*, a London publication, August 20, 1898, we find the opinion expressed that the modern physician is surfeited and embarrassed with the richness of the materia medica that is constantly poured out upon him by the manufacturing chemist. His experiments with new drugs leave no time for practical experience with diseased conditions. The editor says,—

"The present writer has a grievance—a real, determined, angry grievance—against England, Germany, and America. These are the three countries which deluge medicine with physiology, good, bad, and indifferent, but mostly bad; which flood it with literature in the shape of medical books, with no soul of either science or practice in them; and which 'evolute' new remedies, not by the score, but by the thousand annually, not one of which in fifty is worth even so much as a second thought. The inevitable effect of all this upon the average minds in the profession is either to suffocate and so to paralyze them with what appears to be new knowledge, or else to so disgust the practitioner that he makes up his mind never to read at all, and on no earthly consideration whatever to experiment with a new drug. Medicine, in short, is swamped, drowned, stifled, and paralyzed by innumerable exploiters within and without its ranks; exploiters whose only object is the shortest possible cut, not to fame and fortune, but to notoriety and pelf. Now, all this has an exaggerated sound about it. But, indeed and indeed, however exaggeratedly it sounds, it does not express one-tenth part of the miserable truth. The steady practitioner, whose aim is to supply his patients with the very best resources which the science of the times can afford, finds that about half his busy hours are spent in the brain-wearing, and what should be quite unnecessary, operation of separating the precious from the vile."

This seems as applicable to the conditions existing in dentistry as in medicine.

The cause or causes of decay in human teeth may be and are complex and often difficult to satisfactorily explain; but one fact is established in the minds of all true observers,—viz., that decay of the human teeth results from the erosive or chemical action of external agents. Whether decay be regarded as chemico-vital (old theory), chemico-parasitical (Miller), a chemical change produced by minute organisms in the fermentable matter lodged upon and between the teeth (Carr), or whether it is, as some maintain, due solely to bacteria and their products, *all act from and upon the surface of the teeth.*

When Professor McQuillen demonstrated the existence of what he called interglobular spaces,[1] under the enamel and in the dentine of some teeth, it was for a time inferred that decay might in some instances have its beginning within the tooth, in the dentine. But when it was shown that these so-called interglobular spaces were imperfectly calcified, and not decalcified, dentine, the theory of internal decay lost its only support, and it now has but few advocates. All tooth-decay begins at and advances from the exposed surfaces of the teeth, and the work of decalcification moves forward on the line of the tubules just in proportion as the chemical forces acting externally are able to neutralize the vital force which has bound and holds the enamel and dentine as tooth-structure.

The resistance which the tooth interposes to decay-producing agents is wholly from two sources,—1st, the composition, make-up, or vital density of its surface; 2d, the influence of the living pulp in an endeavor to maintain the integrity of the tooth.

Pulp-action in some cases is so vigorous and energetic in opposing the encroachments of decay that new and denser material is deposited not only in the substance of the dentine, but in the pulp-cavity as well. This, however, is the marked exception, and by no means the rule. Pulp-tissue in general, acting for the conservation of the tooth, opposes but feeble resistance to the chemical forces in contact with the surfaces of the teeth. Decay of the teeth, then, may be regarded as a battle between chemical agents and the vitally organized tooth-structure, the strength and activity

---

[1] Czermak, 1850, was the first to describe "interglobular spaces." Tomes (Sir John), 1859, called these "areolar dentine."—Ed.

of the contest depending entirely upon the affinity of the chemical for the elements comprising the tooth and the resistance offered by the consolidated tooth material.

This is strictly accurate in its application to the crowns of devitalized teeth. In teeth with living pulps the chemical action is modified only as the vital forces in the tooth tend to retard resolution. This being true, it is plain that a tooth will be wholly preserved from decay if protected on its exposed surfaces from all chemical action. Cover the crown of a tooth with a gold cap, for instance, embedded in one of the phosphate cements, and the tooth will be freed from all liability to decay, although it has been shown that the phosphate cements are not bacteria-proof. Silver nitrate is advocated as a barrier to the progress of decay; its action, like that of the phosphates, is to mechanically protect the surfaces to which it is applied. How, then, shall we relieve all teeth from liability to decay? Obviously by preventing the permanent lodgement of decay-producing agents, whether they be acids, alkalies, or bacteria, singly or in combination, upon the exposed surfaces of the tooth,—in other words, by giving tooth-tissue destroyers no time to chemically break up the vital combinations existing in enamel and dentine, and resolve their elements into new chemical compounds.

If we consider the condition of decay first encountered, generally induced and fostered by the dark-greenish deposit at the cervical margins, especially of the front teeth in the mouths of children, appearing frequently at two years of age, and continuing on to the age of twelve or fifteen, and even later, we find these mouths veritable crucibles, having in them a mixture of decaying foods, foul odors, natural and artificial acids, bacteria, and heat. It can be no mystery to any that nature's best endeavors are thwarted and completely defeated amidst such surroundings.

What prophylactic treatment shall be instituted and maintained in these and less marked cases? Dr. Williams, in the letter to *Items of Interest*, already alluded to, says, "I have repeatedly pointed out that in my judgment the greatest hope for the future in the saving of human teeth lies in the direction of prevention of decay *by the use of germicides.* [The italics are mine.]

"In my own practice I have relied chiefly upon a strong solution of hydronaphtol in oil of cassia. . . . This I use freely in all cavities, and then, before filling, I use a varnish of Canada balsam in chloroform in which there is a ten per cent. hydronaphtol. My patients use a dentifrice in which hydronaphtol and oil of cassia are the principal germicides. Decay in many instances has been almost entirely arrested. I am, I believe, speaking with all due caution when I say that in my judgment two-thirds of the decay of teeth now going on is preventable."

Whatever of good there may be in these suggestions, they fall far, very far short of meeting the requirements of true dental prophylaxis. Permit an extract from the paper of Dr. Carr before the North Carolina State Dental Society, already referred to:

"Another established fact is that decay of the teeth is due to external causes. The process of destruction is from without inward. . . . Now, then, conceding the fact that the chief cause of caries is the presence of bacteria, it becomes at once our highest duty to apply some remedy for freeing the oral cavity of these destructive organisms, realizing that in a scientifically clean mouth there can be no caries." After setting forth these facts, Dr. Carr attempts a discussion of the irrelevant matter of foods for patients, arriving at the conclusion that "it appears to be out of the question to regulate the diet of our patients, except in case of infants and children whom we can control."

I have made this latter quotation to give emphasis to the erroneousness of this and similar sentiments which crop out from time to time.

The proposition that the dental profession in its present standing, deficient in medical training and lacking in influence, can or should assume to "control the diet of infants and children," trenches on the borders of absurdity, and panders to that spirit of self-adulation already over-cultivated. Had we the power, it would become us to give to every mother and every child a plentiful supply of healthful food, consisting of cereals, vegetables, nuts, fruits, and meats, and thus, and thus only, through diet, lay the foundation for good teeth. No special feeding is demanded for the teeth, neither will such feeding be tolerated by the general system. Foods that will make good bone, muscle, and nerve-tissue are equally good for the proper building of teeth.

It is much more commonly want of vigorous, healthful circulation in the tooth than lack of food-supply which gives rise to poor tooth-structure.

Teeth require use, exercise, stimulation, and, more than all, they require to be kept free from the paralyzing effects of external deposits. Dr. Carr says truly that "in a scientifically clean mouth there can be no decay." To effect this requires something more than germicidal washes, soaps, dentifrices, or even the so-called prophylactic brushes. Frequent friction and vigorous polishing on all crown surfaces will alone accomplish it. This polishing is best effected with a stick—as orange-wood—and a suitable grit,—as fine pumice. Cleansing by such means insures removal of all injurious matter from the surfaces, compels change of environment, stimulates free and healthful circulation within the tooth, and induces the deposit of vitally organized, decay-resisting matter for enamel and dentine. The stimulation resulting from regular and persistent stick-polishing is entirely different from that cleansing which attends the ordinary use of dentifrice and brush.

None of the means commonly employed by patients, as the brush and dentifrices, dental floss and washes, implies *cleanliness* for the teeth, much less freedom from decay; but a regular system of surface-polishing with stick and grit gives positive cleanliness, effecting as it does the absolute removal of all deposits,—solids, semisolids, viscid fluids, and bacteria,—thus placing the tooth in the best possible condition to resist decay. The importance of this method of cleansing and stimulation in the mouths of children, commencing with the deciduous teeth at about two years of age, can scarcely be over-estimated.

It should be continued at intervals of about a week during the period of the temporary teeth, and maintained with equal regularity after the eruption of the permanent ones, gradually extending the time for cleansing to periods of three or four weeks on to adult life. The results from this treatment have proved uniformly beneficial.

Mothers taught the importance of caring for the teeth of children and instructed in this method may be armed with a good porte-polisher, orange-wood and levigated pumice, and have the preservation of their children's teeth in their own keeping.

Since the introduction of the multiplicity of felt and rubber wheels and disks for polishing with power, the hand porte-polishers have fallen into disuse, and none worthy the name are now to be found at the dental depots. A slight modification of the "Jack" porte-polisher, readily obtainable, would meet all requirements in this direction.

Power polishers, whilst justly accorded a place for polishing metallic fillings, are not adapted for reaching the surfaces of many teeth most needing attention, and they are contraindicated in the mouths of children.

Stimulation of pulp-tissue, resulting in improved tooth-structure, is a noticeable and most beneficial result of the process of hand-polishing herein described.

